# Suicidal ideation and behavior among perinatal women and their association with sleep disturbances, medical conditions, and known risk factors

**DOI:** 10.3389/fpsyt.2022.987673

**Published:** 2023-01-19

**Authors:** Bar Arditi-Arbel, Sami Hamdan, May Winterman, Yari Gvion

**Affiliations:** ^1^Department of Psychology, Bar-Ilan University, Ramat Gan, Israel; ^2^Department of Psychology, The Academic College of Tel Aviv-Yaffo, Tel Aviv-Yafo, Israel

**Keywords:** postpartum, suicide, suicidal ideation, suicidal behavior, peripartum, perinatal

## Abstract

**Objective:**

Suicide is considered one of the leading causes of maternal mortality, especially among women with postpartum depression. In the current systematic review, we conducted a qualitative data synthesis of recent studies exploring novel risk factors including sleep disturbances and medical conditions, alongside known and significant risk factors for perinatal suicidality.

**Evidence acquisition:**

We conducted a systematic search of the literature according to PRISMA guidelines on PubMed, PsycNET, and Scopus databases. Search terms were “pregnancy” “OR” “postpartum” “OR” “peripartum” “OR” “perinatal” “OR” “postnatal” combined with the Boolean “AND” operator with “suicide” “OR” “suicidality” “OR” “suicidal ideation” “OR” “suicidal behavior.”

**Evidence synthesis:**

The initial search yielded 1,458 records, of which 51 research reports that met inclusion criteria were analyzed. These 51 studies sampled a total of 45,942 participants. Clinically, sleep disturbance, psychopathology, and social support have been identified as dominant risk factors for suicidal behavior among pregnant and postpartum women, as well as medical conditions and aversive life events.

**Conclusion:**

Monitoring sleep disturbance, depression, and perceived social support is critical given that they are significant risk factors for suicide among perinatal women. Early identification of perinatal women who may be at risk of suicide, although not depressed, is crucial.

**Limitations:**

The use of tools designed to identify depression to identify suicidal risk, fail to identify women who are at risk but who do not suffer from depression. Other methodological limitations are the lack of longitudinal studies and the complexity of examining suicidal behavior in sample studies.

## Introduction

Suicide is a multifactorial phenomenon and a major public health concern that is frequently related to major depression and other mood disorders (1). According to the World Health Organization (WHO) there are around 800,000 suicides a year worldwide (2) yet studies highlight that suicide rate is often underreported (3).

This manuscript aims to present a systemic review that highlights the association of sleep disturbances and medical conditions to suicidal ideation and behavior among perinatal women. Sleep disturbances and medical conditions are common correlates of this population, but their link to suicidal ideations and behaviors have not yet been studied in depth.

Although findings suggest that motherhood protects against suicidality (4), still, suicidal ideation (SI) and suicidal behavior (including plans and suicide attempts) are also common among pregnant and postpartum women. Suicide is among the leading causes of maternal mortality (5, 6) and is a leading cause of death among women with postpartum depression (PPD) (7). Although the concept of PPD is common, today, there is an understanding that, at least in some cases, women face emotional difficulties already during pregnancy which continue into the postpartum period. Indeed, in the last decade, the definition has been expanded and currently it is customary to refer to depression that began during pregnancy as peripartum/perinatal depression (8). Although the perinatal period spans from pregnancy to 1 year after birth, the literature also uses the term perinatal interchangeably with the prenatal/antepartum and postnatal/postpartum periods.

Even though the prevalence of perinatal suicide is lower than in the general population, studies suggest that it is executed through more violent methods (6, 9, 10), thereby indicating high intent and high probability of death from suicide. Khalifeh et al. (10) reported that out of the suicides among women who were in contact with psychiatric services (between 1997 and 2012 in the UK), 2–4% were women in the perinatal period. These findings emphasize the need for early identification and intervention among women at risk in this period. In addition, SI and suicide intent are more common among pregnant and postpartum women (11). Some studies indicate frequency rates ranging between 4 and 15% among women in this period (12), while others have reported 30% in western countries (1). SI is a robust predictor of suicide (13) and in the perinatal period, it is associated with previous SI and/or suicidal attempts (SA) and current mood or anxiety disorders (9, 14).

Over the past 20 years, several systematic reviews have been conducted, of which some focus mainly on SI during pregnancy (15) or throughout the perinatal period (16). Others highlight SA in the postpartum (1, 7) or throughout the perinatal period (6). Three additional research reviews have been published in recent years. While the first two did not examine risk factors specifically (17, 18) the third reviewed studies published up to June 2021 pertaining to risk factors for suicidal ideation and behavior (SIB) among perinatal women (19). However, this review did not address the role of sleep disturbances, problems, or medical conditions on SIB among pregnant and postpartum women. Furthermore, less is known about the extent to which sleep affects SIB in the presence of depressive symptoms and other psychological adversities.

Different studies have found that sleep disturbances are significantly associated with SIB in general (20), and among pregnant (21) and postpartum women (22), in particular. Given that pregnancy and the postpartum period are naturally accompanied by fatigue and lack of quality sleep, it is critical to understand how sleep disturbances during these periods may affect the development of depression and suicide. In addition, studies indicate an association between medical conditions or chronic diseases and SI or SA (23). Since pregnant and postpartum women sometimes face complex medical conditions or chronic illnesses, it is imperative to expand the knowledge of the impact of these conditions on SIB among these women. Our review aims to fill this gap.

The increasing number of studies in the field in recent years (24, 25) emphasizes the importance of further research on the subject and of conducting updated systematic reviews with an emphasis on sleep disturbances and medical conditions. In addition, there is importance in understanding how these two factors moderate or mediate other well-known factors on SIB among perinatal women.

Thus, the current review aims to explore the impact of sleep disturbances and medical conditions on SIB alongside different clinical variables during the perinatal period. We will focus on studies published between 2015 and 2022 (May 15). By deepening the knowledge of the main risk and protective factors for suicidal perinatal women, it will be possible to identify a profile of women at immediate risk and contribute to prevention and intervention programs designed to assist these women.

## Materials and methods

### Information databases and searches

A comprehensive electronic search strategy was applied to identify empirical studies on risk factors for SIB among women in the perinatal period published in peer-reviewed journals. This strategy is in line with Preferred Reporting Items for Systematic Reviews and Meta-Analyses (PRISMA) (26). PubMed, PsycNET, and Scopus databases were searched. Search terms were “pregnancy” “OR” “postpartum” “OR” “peripartum” “OR” “perinatal” “OR” “postnatal” combined with the Boolean “AND” operator with “suicide” “OR” “suicidality” “OR” “suicidal ideation” “OR” “suicidal behavior.” In addition, a manual search in Google Scholar was performed for hitherto unidentified studies.

### Study selection

Studies were eligible if their sample included pregnant or/and postpartum women written in English and presented data regarding a wide range of risk factors associated with SIB among women in the perinatal period. The search revealed a large number of variables, some of which were already included in systematic reviews recently published. We decided to focus on novel ones along with those who can contribute to identifying a profile of perinatal woman in imminent risk for SIB. Therefore papers that focused only on personality characteristics and sociodemographic variables were removed from the final stage of the selection.

The studies included in this review used various tools to measure SIB. One common tool used is the Edinburgh Postnatal Depression Scale (EPDS) (27), which assesses the current level of depression symptoms among perinatal women. The tenth item of the EPDS assesses self-harm behavior (“the thought of harming myself has occurred to me”). Given that the research literature is divided as to whether to include self-harm behavior as part of SIB, we decided to exclude studies that specifically addressed self-harm without suicidal intent (NNSI) as opposed to studies that reported thoughts of self-harm as part of the use of the standard EPSD questionnaire. The latter were included as studies that report the range of SIB and in which the reported findings were written according to the definitions that appear in the studies themselves.

To review the most recent and up-to-date findings, we searched published studies dating from January 1, 2015 to May 15, 2022. Case studies, reviews, book chapters, conference papers, and incomplete studies were excluded. Three psychologists (two Ph.D.s, one Ph.D. candidate) and one graduate student in psychology examined both the consistency of the search and the suitability of the studies considering the inclusion and exclusion criteria.

### Data analysis

Given that studies on suicide risk factors among women in the perinatal period are highly varied, with different sample types, study designs, and measurement tools, they could not be combined into a single meta-analysis study. Therefore, we conducted a systematic review whereby studies were first categorized based on risk factors (e.g., sleep disturbances, medical conditions, interpersonal factors, etc.) and then summarized by highlighting common features in each group.

## Results

**FIGURE 1:**
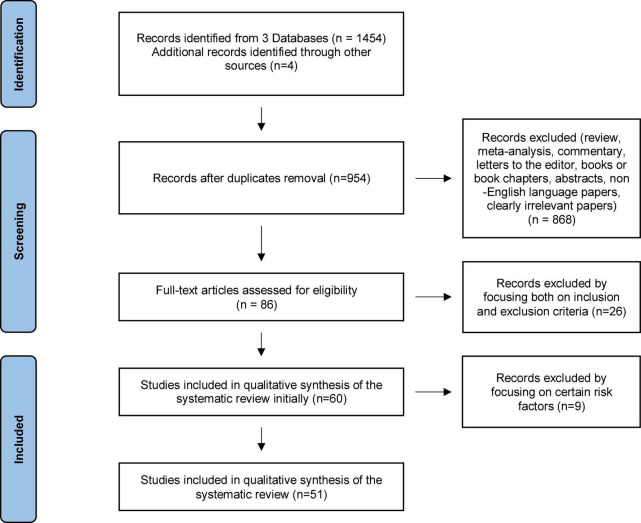
PRISMA flowchart of the systematic search.

The initial search conducted in electronic databases yielded *n* = 1,458 citations, as reported in the PRISMA flowchart (Figure 1). Based on both inclusion and exclusion criteria, 51 research studies and a total of 45,273 participants were identified and included in the systematic review (see flowchart in Figure 1). In what follows, we present the literature review results, which have been sub-grouped according to common risk factor themes. First, we will address the novel risk factors studied: sleep disturbances and medical conditions. Then we will review the latest studies relating to other main risk factors. A detailed description of reviewed studies is presented in Table 1.

**TABLE 1:** Distribution of the 51 relevant selected studies including the references, number of participants, aims, suicidality outcomes, and psychometric instruments.

	References	Number of participants	Aims	Suicidality outcomes	Psychometric instruments used to measure suicidality
1	Abdelghani et al. (62)	*n* = 835	To address the current suicide risk, and evaluate its correlates of among pregnant women in Egypt	CRS	BSS
2	Akram et al. (49)	*n* = 547	To explore postpartum depression as a predictor of suicidal ideation and to find if perceived social support had a moderator effect in this scenario among new mothers with hearing loss	SI	SIDAS
3	Alhusen et al. (59)	*n* = 166	To examine the prevalence of suicidal ideation and comorbid depressive symptomatology during pregnancy, and to identify the risk factors for suicidal ideation during pregnancy in a low-income, predominantly African American sample of pregnant women	SI	EPDS
4	Anbesaw et al. (34)	*n* = 423	To explore the prevalence of suicidal ideation and associated factors among pregnant women attending antenatal care in Jimma, Ethiopia	SI	CIDI
5	Bao et al. (35)	*n* = 100	To determine if sleep quality and decision-making dysfunction ability are associated with suicidal ideation in prenatal women with depression compared to prenatal women without depression	SI	EPDS
6	Belete and Misgan (43)	*n* = 988	To assess the prevalence and associated factors of suicidal behavior (suicidal ideation, plan, or suicide attempt) in postpartum mothers	SB	MINI
7	Belete et al. (48)	*n* = 762	To assess the prevalence and factors associated with suicide ideation and attempt among pregnant women attending antenatal care services at public hospitals in southern Ethiopia	SI and SA	CIDI
8	Coker et al. (36)	*n* = 842	To (1) compare the sensitivity of self-rated and clinician-rated scales in identifying maternal thoughts of self-harm in the postpartum period; To (2) examine obstetrical and neonatal outcomes potentially associated with thoughts of self-harm, and to (3) examine prenatal and postnatal characteristics associated with postpartum suicidal ideation as potential risk factors	SI	BDI, EPDS, BDI
9	de Avila Quevedo et al. (68)	*n* = 706	To assess the suicide risk in women who experienced depressive or mixed episodes of mood change during the postpartum period and to determine which disorder is more related to suicide risk in the same period	SR	MINI
10	Doi and Fujiwara (73)	*n* = 8,074	To examine the combined effect of maternal adverse childhood experiences and maternal age on self-harm ideation among postpartum women	SI	EPDS
11	du Toit et al. (54)	*n* = 280	To assess suicidality and associated factors during pregnancy and the postpartum period amongst women with known psychiatric diagnoses	Suicidality (suicidal ideation, plan, or attempt)	MADRS, MINI, and a clinical assessment interview
12	Castro e Couto et al. (63)	*n* = 255	To evaluated the prevalence of suicidality during the second trimester of pregnancy	CSR and lifetime SA	BDI, EPDS, MINI
13	Faisal-Cury et al. (56)	*n* = 358	To evaluate the association between mother–child bonding at 6–9 months after birth and suicidal ideation	SI	PHQ-9
14	Fellmeth et al. (69)	*n* = 568	To report data on suicidal ideation among migrant and refugee women living on the Thailand–Myanmar border	SI	SCID
15	Friedman et al. (42)	*n* = 3,372	To examine the association between migraine and suicidal ideation among a cohort of pregnant women	SI	PHQ-9
16	Garman et al. (70)	*n* = 384	To assess the association between depressive symptoms and suicidal risk over time among perinatal women at risk for depression antenatally, and assess modifying effects of age, perinatal stage and depressive symptom trajectory	SIB	MINI
17	Gelaye et al. (31)	*n* = 641	To examine the independent and joint relationships of poor subjective sleep quality, and antepartum depression with suicidal ideation among pregnant women	SI	PHQ-9
18	Gelaye et al. (32)	*n* = 1,298	To evaluate the independent and combined associations of maternal self-reported poor sleep quality and antepartum depression with suicidal ideation during the third trimester	SI	PHQ-9
19	Gressier et al. (51)	*n* = 1,439	To assess risk factors associated with SA in pregnancy and in the post-partum period in women with mental health disorders	SA	Retrospective evaluation of SA
20	Gross et al. (77)	*n* = 620	To investigate the impact of Military sexual trauma (MST) on risk of depression and suicidal ideation (SI) during and after pregnancy	SI	EPDS
21	Islam et al. (66)	*n* = 426	To examine the association of experiencing physical, psychological, and sexual IPV after childbirth on postpartum suicidal ideation; and whether postpartum depression and self-esteem act to mediate or moderate the relationship between IPV and postpartum suicidal ideation	SI	EPDS
22	Jones et al. (65)	*n* = 599	To examine suicidal ideation in the post-partum period, comparing women with detectable and undetectable efavirenz at 32 weeks of pregnancy	SI	EPDS
23	Kalmbach et al. (29)	*n* = 267	To explore the relationships of depression and suicidal ideation with insomnia, short sleep, and nocturnal rumination in mid-to-late pregnancy	SI	EPDS
24	Kalmbach et al. (30)	*n* = 39	To explored associations among insomnia, nocturnal cognitive hyperarousal, and nocturnal perinatal-focused rumination with suicidal ideation in perinatal women with depression	SI	EPDS
25	Kalmbach et al. (28)	*n* = 99	To estimated survey-assessed DSM-5 insomnia disorder rates in pregnancy, and described associated sociodemographics, and sleep-wake and mental health symptoms	SI	EPDS
26	Knettel et al. (38)	*n* = 200	To examined patterns of suicidal ideation and associated factors among pregnant and postpartum women living with HIV in Northern Tanzania	SI	EPDS, PHQ-9
27	Kubota et al. (60)	*n* = 430	To elucidate the foreseeable risk factors for suicidal ideation among Japanese perinatal women	SI	EPDS
28	Kugbey et al. (74)	*n* = 214	To examined the prevalence and correlates of prenatal depression, anxiety, and current suicidal behaviors among pregnant women in the Volta Region of Ghana	SB	Single items examining both lifetime and current SB developed by the researchers
29	Maré et al. (61)	*n* = 748	To assess the prenatal and postnatal prevalence of suicidal ideation and behavior and sociodemographic, psychosocial, and psychiatric correlates, *via* stratified analyses, of suicidal ideation, and behavior presence and severity	SIB	MINI
30	Martini et al. (53)	*n* = 306	To investigate predictors of peripartum suicidality and potential maternal and infant outcomes of PS	PS	BSI, CIDI-V, EPDS
31	Molla et al. (37)	*n* = 504	To assess the prevalence and associated factors of suicidal behavior among pregnant mothers to integrate mental health care, particularly suicide, with maternal management	SB	SBQ-R
32	Muzik et al. (52)	*n* = 116	To extend the understanding of postpartum SI in the context of CM. the first aim is to assessed the presence and severity of SI with frequency statistics. The second aim is to investigate associations between potential risk and resilience factors and the presence or severity of postpartum SI	SI	SI Sub-scale of the PDSS
33	Onah et al. (45)	*n* = 376	To investigated the prevalence and associated psychiatric and socio-economic contextual factors for Suicidal ideation and behavior among pregnant women living in low resource communities in South Africa	SB, lifetime SA, frequency of SI, and intention of suicidal act	MINI Plus
34	Palagini et al. (21)	*n* = 62	To explore sleep reactivity in pregnant women and its relations to prenatal symptoms of insomnia, depression, anxiety, and suicidality	SI	EPDS
35	Palfreyman (47)	*n* = 1,000	To assess the prevalence of antenatal depression and lifetime- and current-pregnancy suicidal ideation and/or behavior. To (2) understand what is the relationship between depression and suicidal ideation and behavior among antenatal population and to (3) identify what correlates with depression and suicidal ideation and behavior	SIB	C-SSRS, EPDS
36	Peltzer (39)	*n* = 580	To investigate the prevalence of suicidal ideation and its associated factors in postpartum HIV-positive women in South Africa	SI	EPDS
37	Redinger et al. (55)	*n* = 649	To examine the rates of thoughts of self-harm across pregnancy in a longitudinal South African cohort, and to investigate factors associated with the onset and persistence of TSH, as well as the relationship between TSH, depression, and/or anxiety	Thoughts of self-harm	EPDS
38	Rodriguez et al. (64)	*n* = 673	To estimate the prevalence of and identify risk factors for Suicidal ideation among pregnant with HIV in rural SA	SI	EPDS
39	Rodriguez et al. (40)	*n* = 403	To identify risk factors for suicidal ideation in women living with HIV during pregnancy, and to assess their evolution, including continued suicidal ideation into the postnatal period, as well as the emergence and cessation of postnatal suicidal ideation	SI	EPDS
40	Rurangirwa et al. (50)	*n* = 921	To investigate the prevalence of non-psychotic mental health disorders and the association between exposure to all forms of intimate partner violence (IPV) during pregnancy and MHDs	SI	MINI
41	Shamu et al. (76)	*n* = 842	To investigated the association of postnatal depression (PND) and suicidal ideation with emotional, physical, and sexual IPV experienced by women during pregnancy	SI	One question about having SI in their lifetime and in the last month
42	Sit et al. (22)	*n* = 628	To determine whether the known risk factors for suicidal symptoms in adults with and without mood disorders also applied to women after childbirth	SI	EPDS
43	Supraja et al. (46)	*n* = 462	To estimate the prevalence of suicidality during early pregnancy and identifying associated and predictive factors among women attending a public health antenatal clinic	Suicidality (suicidal ideation, planning, and attempts)	EPDS, SBQ-R
44	Tabb et al. (75)	*n* = 701	To identify whether exposure to intimate partner violence was associated with increased risk for suicidal ideation among low-income postpartum women from São Paulo, Brazil.	SI	CIS-R
45	Takegata et al. (57)	*n* = 243	To identify women with thoughts of self-harm preceded by suicidal ideation, during the perinatal period, on cluster analysis, and to clarify their psychological correlates	SI	EPDS
46	Weng et al. (33)	*n* = 3,867	To (1) examine the occurrence of suicidal ideation, depression, and anxiety in pregnant women from the prenatal to postpartum periods. To (2) investigate the risks of suicidal ideation, depression, and anxiety among perinatal women who are exposed to tobacco, including pre-pregnancy smoking and secondhand smoke; and to (3) explore whether associations between women’s exposure to tobacco and risks for suicidal ideation, depression, and anxiety differed according to the women’s characteristics	SI	EPDS
47	Zewdu et al. (41)	*n* = 422	To determine the magnitude of suicidal ideations in Gondar town among HIV positive perinatal women	SI	CIDI
48	Zhang et al. (67)	*n* = 1,825	To explored the roles of different experiences of childhood abuse in suicide ideation during pregnancy in eastern cultures	SI	PHQ-9
49	Zhang et al. (58)	*n* = 432	To investigate the prevalence of suicidal ideation in the third trimester and associated predictors including psychological factors	SI	EPDS
50	Zhong et al. (72)	*n* = 1,517	To examine the concordance of the two suicidal ideation items among pregnant Peruvian women	SI	EPDS, PHQ-9
51	Zhong et al. (71)	*n* = 2,964	To examine the association between exposure to childhood abuse and suicidal ideation among pregnant women	SI	PHQ-9

SI, suicidal ideation; SA, suicide attempt; SB, suicidal behavior; SIB, suicidal ideation and behavior; CRS, current suicide risk; SR, suicide risk; PS, perinatal suicidality; BDI, beck depression inventory; BSI, brief symptom inventory; BSS, beck suicidal ideation scale; CIDI, composite international diagnostic interview; CIDI-V, composite international diagnostic interview for women; CIS-R, clinical interview schedule-revised; C-SSRS, Columbia-suicide severity rating scale; EPDS, Edinburgh postnatal depression scale; HDRS, Hamilton rating scale for depression; MADRS, Montgomery Asberg depression rating scale; MINI, mini international neuropsychiatric interview; MINI Plus, the expanded mini-international neuropsychiatric interview; PDSS, postpartum depression screening scale; PHQ, patient health questionnaire; PHQ-9, nine-item patient health questionnaire; SBQ-R, suicidal behavior questionnaire; SCID, the structured clinical interview for DSM-5; SIDAS, suicidal ideation attributes scale.

### Poor sleep quality and sleep disturbances

Fatigue and sleep disturbance are well-known risk factors for suicidality (20), and are very common among pregnant and postpartum women. However, it is surprising that despite this high prevalence, there has only been an increase in the number of studies on the subject in recent years. Eleven studies assessed the association between sleep quality or sleep disturbance and SIB among a total of 8,266 participants.

A recent cross-sectional study among 99 pregnant women indicated that insomnia was associated with depression and suicidality (28). Another cross-sectional study explored the relationship between depression and SI, insomnia, short sleep, and nocturnal rumination during pregnancy. The study was part of a large randomized controlled trial (RCT) in a six-hospital health system in metropolitan Detroit. The study sample included 267 pregnant women in the late second and early third trimester recruited from obstetric clinics which completed online surveys. The researchers used a four-group comparison based on insomnia status and high/low rumination. They found that pregnant women with high rumination and insomnia suffered from higher rates of depression (35.6%) and SI (17.3%) compared to pregnant women who slept well (1.2% depressed, 4.9% suicidal) (29). Following these findings, a longitudinal study conducted among 39 women with perinatal depression found that the highest risk of SI was among women who have insomnia and nocturnal cognitive hyperarousal (30).

Gelaye et al. (31) and Gelaye et al. (32) conducted cross-sectional studies in Peru to evaluate the relationships between self-reported sleep quality, antenatal depression, and SI during pregnancy and found similar results. In the first study, 641 pregnant women were recruited from an ongoing Pregnancy Outcomes, Maternal and Infant Study cohort. Over 15% of the sample reported SI during pregnancy and poor subjective sleep quality. After adjusting for confounders and depression, the researchers also found that poor subjective sleep quality was associated with 67% increased odds of SI among the study population. The second study assessed 1,298 pregnant women visiting prenatal clinics for sleep quality, depressive symptomatology, and SI. It was found that pregnant women who reported poor sleep quality and depression were also more likely to suffer from SI than pregnant women who did not report these risk factors. Even after adjusting for antenatal depression, poor sleep quality was still associated with SI. Weng et al. (33) conducted a large cross-sectional study at five hospitals in Taiwan and found in a multivariate analysis that pregnant women who reported poor sleep were also significantly more likely to experience SI. Similar findings were found in an Ethiopian cross-sectional study among 423 pregnant women (34).

Palagini et al. (21) examined the relationships between sleep reactivity (the degree to which the person is vulnerable to sleep disturbance when exposed to stress) in pregnancy and prenatal symptoms of insomnia, depression, anxiety, and suicidality. They evaluated 62 pregnant women at the gynecological unit at the University of Pisa, Italy. The sample was a sub-sample from a larger study that investigated the outcomes of prenatal sleep disorders and psychopathology outcomes between 2017 and 2018. The researchers compared two participating groups, with the first including a group of women with high stress-related sleep reactivity and the second including women with low sleep reactivity. They found that the first group reported more depression and anxiety and were more likely to endorse SI than the second group. Lastly, Bao et al. (35) found that SI correlated with subjective sleep quality, duration, and daytime dysfunction among 100 pregnant women in the third trimester. It was also found that women who suffered from depression and SI indicated significantly poorer sleep quality than women who suffered from depression without SI.

As for studies conducted among postpartum women, Sit et al. (22) conducted a secondary analysis to determine whether the known risk factors for suicidal symptoms in adults, such as sleep disturbance, the experience of abuse in childhood or adulthood, and anxiety symptoms, also applied to women after childbirth. The analysis was based on a large study designed to examine the outcomes of unipolar major depression in women during the first postpartum year. The analysis included 628, 4–6 weeks postpartum women with depression-positive diagnoses. The researchers noted a significant association between current sleep disturbance and SI among women with postpartum depression without a history of childhood physical abuse, as opposed to women with a specific abuse history. The researchers suggested that it is possible that the small number of subjects in each subgroup contributed to the lack of statistical significance. Like the relationship found among pregnant women, a study that evaluated 842 postpartum women with neuropsychiatric illnesses reported that those having more difficulty sleeping were also significantly more likely to endorse SI (36).

### Medical conditions

Women face different medical conditions, some chronic, either of their own or their fetus/baby during pregnancy and the postpartum period, which may affect them mentally. Seven studies [a total of 6,747 participants] have addressed the association between medical conditions and SIB in pregnant and postpartum women. Of them, six studied the impact of the woman’s medical condition on her emotional state, while one addressed the newborn’s medical condition on the mother’s mental state.

An Ethiopian cross-sectional study was conducted among 504 pregnant mothers and found that having a history of chronic medical illness as tuberculosis, HIV, and hypertension was associated with suicidal behavior (SB) in pregnancy (37). A longitudinal cohort study conducted in Northern Tanzania among 200 pregnant women with HIV (diagnosed prior to or during current pregnancy) included two measurement points, one during pregnancy and one approximately at the 6-month postpartum point. The results indicated a significant decrease in the rate of SI from pregnancy (12.8%) to postpartum (3.9%) among participants. It was found that a lack of clarity regarding the HIV status of the child’s father, negative attitudes toward HIV medication, internalized HIV stigma, and shame was associated with SI (38). Two additional studies found a high stigma toward HIV as a risk factor. The first was a cross-sectional survey conducted among 580 HIV-positive postpartum women in 48 health facilities in South Africa. Results revealed that a sexually transmitted infection diagnosis was also associated with SI during the postpartum period (39). The second study aimed to identify risk factors for SI among women living with HIV during pregnancy and postpartum. Data were extracted from 681 women participating in larger, longitudinal prevention of mother-to-child transmission clinic-randomized controlled trial (RCT). Disclosure of HIV status to a partner was found to have a protective effect (40). In another study conducted among 422 HIV-positive perinatal Ethiopian women, non-disclosed HIV status was associated with SI (41).

One study referred to migraine headaches as a risk factor among pregnant women. The cohort consisted of 3,372 pregnant women treated at prenatal care clinics in Peru and data was based on information collected in a larger study. 16% of participants endorsed SI, whereas 26.2% reported depression. Women who experienced migraines were at significantly higher risk for experiencing SI than women who did not suffer from migraines, even after adjusting for various confounders, including depression. It was further found that among women without depression, the migraine experience significantly increased the risk of experiencing SI compared to women without migraines. Furthermore, women who experienced both depression and migraines were at a higher risk than women who did not experience any of the risk factors (42). Lastly, in a cross-sectional study in two health centers in Ethiopia in which 988 postpartum women were assessed, newborn illness was significantly associated with SB (planned or attempt) (43).

### Interpersonal factors

#### Social support

Social support is known as a dominant resilience factor when dealing with a stressful situation; therefore, during pregnancy and postpartum, this support is essential for both the mother’s and family’s adjustment (44). In 13 studies, social support was found to be a central risk factor for depression and suicidality during pregnancy and postpartum, among a total of 7,752 participants. Four cross-sectional studies were conducted among pregnant women. The first, with a cohort of 376 pregnant women living in low-income communities in South Africa, investigated the prevalence and the associations between psychiatric and socioeconomic factors and SIB. The results revealed that when the perception of social support from a significant other increased, pregnant women reported reduced SIB rates (45). The second study was nested within the Prospective Assessment of Maternal Mental Health study. The study investigated the prevalence of suicidality among 426 pregnant women treated at a public health antenatal clinic in India. Women who indicated having SI also perceived poor social support, while higher perceived social support was associated with decreased odds of suicidality during pregnancy (46). The third study was conducted among 1,000 urban Sri Lankan pregnant women and found that a lack of perceived social support was associated with SIB (47). Lastly, the fourth study was conducted in southern Ethiopia among 762 pregnant women attending antenatal clinics at public hospitals. The researchers reported that social support was the only variable associated with SA while various variables were linked to SI in pregnancy (48).

Four more cross-sectional studies were conducted among postpartum women. A recent Pakistani study aimed to find whether perceived social support moderated the relationship between PPD and SI among new mothers with hearing loss. The sample included 547 2–6-week postpartum mothers recruited from maternity clinics; 272 (49.7%) were with hearing loss. Perceived social support was an independent variable explaining more than 60% of the variance in SI and a negative and significant predictor of SI among postpartum women with hearing loss. It was also found that the interaction between PPD and perceived social support negatively affected SI (49).

Furthermore, two cross-sectional studies among postpartum women were conducted in Africa. Rurangirwa et al. (50) indicated that poor social support was associated with experiencing non-psychotic mental health disorders, including SI. The sample was composed of 921 postpartum women from Rwanda, most of whom were of low socioeconomic status. The second study included 580 postpartum HIV-positive women from South Africa and found that a lack of social support and a lack of the father’s support (not accompanying the woman to antenatal care) were associated with SI (39). Finally, a large study including 1,439 hospitalized women with different psychiatric pathologies found that poor social support was associated with more SA in this population. The study was based on a database collection based on clinical interviews with the patients and psychiatrists and medical records from the French Network of Mother-Baby Units (51).

Five longitudinal studies assessed the relationship between social support and SI. One study was conducted among 200 Tanzanian pregnant and postpartum women diagnosed with HIV. The results indicated low social support and low support from the child’s father were significantly associated with SI (38). Another longitudinal study included 116 postpartum mothers with a history of childhood maltreatment and non-psychiatrically diagnosed. The authors found that perceived family support was only related to SI severity at 4 months postpartum (52). In a longitudinal study conducted among 306 German women, the researchers found that high social support significantly reduced the odds of perinatal suicidality at weeks 22–24 gestation (53). A longitudinal study among 430 perinatal Japanese women found that good quality social support was a protective factor (54). Lastly, a longitudinal study in urban South Africa among 649 pregnant women it was found that practical support and a good marital relationship reduced the risk of self-harm in early and late pregnancy (55).

#### Mother-baby relationship

Only three studies, with a total of 1,033 participants, examined the relationship between the nature of the mother-infant relationship and SIB in the perinatal period. A cross-sectional study conducted in Brazil among 358 low-income postpartum women found an association between mother-child bonding impairment and SI even after controlling for postpartum depression (56). In another longitudinal study among 243 perinatal women from Japan it was found that feelings of anger, lack of affection, and rejection toward the baby were associated with thoughts of self-harm (57). Finally, one recent study examined the relationship between the mother’s attachment style and SI. The study was conducted among 432 women in the third trimester of pregnancy. The results indicated that women who reported SI were also characterized by an insecure attachment style, as well as attachment avoidance and anxiety (58).

### Psychopathology

Thirty-six studies of 26,035 participants reported present or past psychopathology as a risk factor for suicidality during pregnancy and the postpartum period. Most studies, reported that present or past depression (21, 32, 34, 35, 40–42, 46, 48, 49, 51, 53–67), anxiety (34, 38, 45, 48, 53, 58, 63), and past suicidality (45–48, 53, 54) are significant risk factors for suicidality during pregnancy and the postpartum period. While two referred to psychiatric comorbidity as a significant risk factor (22, 68), two studies referred to family psychopathology as such a factor (47, 68). These findings align with the literature on the strong relationship between these risk factors and suicide in general. For example, among 748 perinatal women the antenatal SIB prevalence was 19.9% and postpartum 22.6%. PTSD independently predicted the presence of SIB at 6 months postpartum, while depression independently predicted SIB in the antenatal and postpartum periods (61). A study conducted among 1,000 antenatal Sri Lankan women found that a history of suicidality increased the risk for SIB in the current pregnancy by times 11 (47).

In a cross-sectional study conducted among 255 pregnant Brazilian women during their second trimesters in prenatal care, 23.53% of the sample was identified as being at various levels of suicide risk. Additionally, it was found that antenatal depression, lifetime bipolar disorder, and any current anxiety disorder are risk factors for suicide (63). Another more recent cross-sectional study among 267 pregnant women in the late second trimester explored the relationships between depression and SI, sleep measures, and nocturnal rumination (i.e., cognitive activity and arousal when trying to fall asleep). Results indicated that SI is associated with negative perinatal-focused rumination and depression among participants (29). A longitudinal study among 39 women with perinatal depression found similar findings in late pregnancy and early parenting (30). In a longitudinal study among 706 Brazilian postpartum women, de Avila Quevedo et al. (68) assessed suicide risk in postpartum women who experienced mood disorders during the postpartum period. Suicide risk was associated with chronic mood disorder, depression, anxiety disorders, and mixed episodes in the postpartum period.

Another quantitative descriptive study among women with known psychiatric diagnoses during the perinatal period was conducted at two maternal mental health clinics in South Africa. Two hundred eighty women participated in the study, with the most common psychiatric diagnosis being MDD and the prevalence of suicidality 24%. Results revealed that women who had previously attempted suicide had a significantly higher risk of experiencing suicidality during their pregnancy. Additionally, it was found that MDD and borderline personality traits were positively associated with suicidality (54). Similarly, a cross-sectional analysis of 736 low-income pregnant women found a strong association between depression and SI; however, it also noted that 35% of women with SI did not meet the criteria for elevated depressive symptoms. A study on the Thailand–Myanmar border indicated that most women who reported SI did not have severe depression (69). These findings contribute to the debate on including suicidality items on depression screens.

Furthermore, a secondary analysis was conducted to assess the longitudinal association between depressive symptoms and suicidal risk over time. Three hundred eighty-four adult pregnant women were recruited from two antenatal clinics in an informal settlement in South Africa. Participants were followed up at 8 months gestation and 3-month and 12-month postpartum. In general, the results suggest a relationship between the variability of the severity of depression over time and the variability of the severity of suicide risk over time. However, this relationship is maintained only if the severity of the depression had decreased. In addition, the longitudinal association was not maintained among older or more chronically depressed perinatal women. Considering the results, the researchers suggest that depression and suicide are overlapping and independent phenomena. These results highlight the importance of assessing symptoms of depression and suicide separately (70).

The use of Substances has also been investigated in some studies. Two studies examined the effect of alcohol use and/or smoking as risk factors for suicide. The first study enrolled 1,439 hospitalized women with different psychiatric pathologies, of which 37.5% reported smoking during pregnancy and 8.4% reported alcohol use. An independent association between SA in pregnancy and alcohol use and smoking was found (51). The second study was conducted by Weng et al. (33) at five hospitals in Taiwan. Among other things, the researchers wanted to examine the occurrence of SI, depression, and anxiety among perinatal women exposed to tobacco smoke. In general, the findings suggest a positive association between secondhand smoke exposure and perinatal depression and SI. In addition, they found that women exposed to smoke were at risk for SI during the second and third trimesters more than women not exposed to smoking.

### Life events

The literature highlights the relationship between aversive life events and mental distress, including depression and suicidal tendencies among women during the perinatal period. Studies are divided into two groups: the first addressing aversive events in childhood and the second focusing on aversive events in adulthood.

#### Aversive events in childhood

Eight studies among a total of 16,418 participants examined aversive events in childhood as risk factors. A recent prospective-longitudinal study conducted among 306 pregnant women treated at gynecological outpatient settings in Germany explored the predictors of perinatal suicidality and potential maternal and infant outcomes of perinatal suicidality. The study consisted of seven evaluation points, three during pregnancy, and four during postpartum. Its results indicated that perinatal suicidality was more likely among women with a history of childhood abuse or rape (53). Similar findings were found in two large cross-sectional studies investigating the effect of childhood abuse experiences on SI during pregnancy. The first was conducted among 1,825 pregnant women in their third trimester of pregnancy treated at a prenatal clinic in China between 2017 and 2018. It was found that women who experienced childhood abuse (8.4% of the sample had experienced some form of childhood abuse) were at high risk for suicide ideation. In addition, women who experienced childhood abuse and were depressed were more at risk than women who did not report these risk factors (67).

The second study included 2,964 pregnant women treated at prenatal clinics in Peru between 2012 and 2014 who had enrolled in an ongoing cohort study. Over 70% of participants reported experiencing a type of childhood abuse; the prevalence of antepartum SI was 15.8%. Results revealed that women who reported childhood abuse were at higher risk for SI even when confounders were adjusted. The odds for SI increased for women who were also depressed compared to women who were depressed but did not experience childhood abuse. Furthermore, women who experienced sexual and physical abuse during childhood had much higher odds of SI. A positive relationship between the prevalence of SI and the number of childhood abuse events has also been found (71). A cross-sectional study, which was part of the cohort study in Peru, found that among the 1,517 women who took part in the study, those with SI were more likely to have experienced childhood abuse (72). Unlike most studies, a secondary analysis among 628 depressed mothers found that compared to physical abuse, a history of sexual abuse did not significantly affect the frequency of self-harm thoughts among the study population. The researchers pointed to methodological limitations that could have influenced this finding (e.g., the use of tools not designed to identify childhood abuse) as it contradicts other findings in the literature reporting a strong link between sexual abuse and SI (22).

Muzik et al. (52) conducted a longitudinal study across 18-month postpartum women as part of the Maternal Anxiety during the Childbearing Years study, to expand the understanding of postpartum SI in the context of childhood maltreatment (CM). The sample study comprised 116 postpartum mothers with CM histories and non-psychiatric diagnoses. Of these, roughly 25% experienced SI during the study. Associations between SI presence and severity of CM were found at 4 months postpartum. Additionally, shame about CM was significantly related to the presence and severity of SI among the study participants. At 12- and 18-month postpartum, shame continued to be significantly associated with SI severity. An Ethiopian cross-sectional study found that a history of rape was related to SB among 988 postpartum women (43). Finally, a Japanese cross-sectional study was conducted among 8,074 postpartum women to investigate the relationship between adverse childhood experiences (e.g., parental death, parental divorce, parental mental illness) and self-harm ideation. Results revealed that adverse childhood experiences are a risk factor for self-harm ideation and that younger postpartum women with three or more adverse experiences were significantly more likely to report self-harm ideation compared to older women with no adverse experiences (73). In general, the findings highlight the severe and lasting psychological effects that childhood experiences can have on women during pregnancy and after childbirth.

#### Aversive events in adulthood

We found 19 studies among a total of 12,920 participants exploring the relationship between the experience of aversive events in adulthood and suicide ideation or behavior during the perinatal period, of which most cited intimate partner violence (IPV) as a possible risk factor. One study examined the history of trauma among perinatal women on the Thailand–Myanmar border and found that it was associated with SI after controlling for all other variables (69). In a cross-sectional study conducted among low-income pregnant women, 20% of the 166 participants receiving prenatal care at a university obstetrical clinic, reported experiencing IPV during the current pregnancy (59). In two studies conducted in Ethiopia among pregnant women, IPV was found as a significant predictor of SI (34, 37). Similar findings were found in a study among pregnant Egyptian women in which an association was found between IPV and current suicide risk (62).

Another cross-sectional study among 376 pregnant women in Hanover Park (a low-income suburb within Cape Town) revealed that women who experienced IPV were twice as likely to report SIB as opposed to those who did not experience IPV (45). Among 1,000 pregnant women from urban Sri Lanka, it was found that a woman who experienced any form of IPV in a current partnership was four times as likely to report SIB in her current pregnancy (47). Similar findings were found in a study among 214 pregnant women in Ghana (74). A cross-sectional study within a prospective cohort study among 426 pregnant women treated at an antenatal clinic at the Government Referral Hospital in India, indicated that SI in pregnancy was associated with IPV or any other form of domestic violence (46).

In five studies, experiencing IPV was a risk factor for postpartum SI. Among 701 low-income postpartum women from primary care clinics in Brazil, IPV was a significant risk factor subsequently, 70% of the participants who experienced SI also experienced IPV (75). Similar findings were found in a cross-sectional study among postpartum women in Peru (72). Another cross-sectional study was conducted in six public clinics in low-income urban areas of Zimbabwe among 842 6-week postpartum women. Results revealed that postpartum SI was associated with emotional IPV during pregnancy. Other types of IPV (physical, sexual) were not found to be directly related unless combined with emotional IPV (76). A recent cross-sectional study among 426 postpartum women examined the relationships between different types of IPV after childbirth and postpartum SI. Additionally, PPD and self-esteem were examined as possible mediators and/or moderators. In general, it was found that subjects who experienced SI were also more likely to report an IPV postpartum experience. Among 68.6% of those who experienced physical IPV (50% experienced sexual IPV and 48.3% experienced psychological IPV) SI was also reported. It was also found that the effect of physical IPV on postpartum SI was moderated and mediated by postpartum depression and maternity self-esteem (66). Rurangirwa et al.’s (50) study found that all forms of IPV (sexual, psychological, physical, and controlling behavior) were significantly associated with SI. The study was conducted in Rwanda among 921 postpartum women of low socioeconomic status.

Three other studies—one cross-sectional and two longitudinal—examined these relationships among HIV-positive women. The first longitudinal study sample included 588 women with HIV. 19% of participants reported SI 12-month postpartum when higher levels of physical and psychological IPV were found among these women at that stage (65). Similar findings were found in the second longitudinal study, which drew its data from a larger RCT longitudinal study. The study sample included 681 pregnant HIV-positive women who enrolled at baseline, with 59% completing the last study point at 12-month postpartum. A relationship between SI and increased psychological and physical IPV was found. Physical IPV also significantly increased the odds of experiencing SI at baseline and at 1-year postpartum (40). The last study among women with HIV was a cross-sectional study conducted in rural South Africa among 673 pregnant women. IPV was a risk factor for SI, and an interaction between physical IPV and stigma were also found (64).

A longitudinal study was conducted among pregnant and postpartum army veterans to understand the effect of military sexual trauma on risk for depression and SI during the perinatal period. Data was taken from a 3-year longitudinal study. Six hundred and twenty veterans took part during pregnancy, while 452 of them were also interviewed after delivery. Higher pre- and postpartum symptoms of depression and SI were found to be associated with military sexual trauma (77). Lastly, three studies indicated that a previous perinatal loss may be another possible risk factor for perinatal suicidality (34, 51, 62).

## Discussion

This review aimed to present and highlight the contribution of sleep disturbances and medical conditions alongside known and significant risk factors to suicidal ideations and behaviors among perinatal women. In addition, we aimed to explore how these two factors moderate or mediate the well-known factors on SIB among perinatal women.

This was a comprehensive review of 51 studies sampled a total of 45,942 participants. Although several previous reviews have been conducted in the field, they did not focus on the role of sleep disturbances and medical conditions in perinatal suicidality. Increasing knowledge about these variables and their relationships with other known risk factors is important in order to identify a possible profile of women at risk for imminent suicidal behavior.

In what follows, we will first summarize the main findings of our review and then highlight methodological limitations pertaining to research on suicidality among perinatal women.

In general, it is evident that in recent years research has begun to focus on the link between sleep disturbance, depression, and SIB. Eleven studies, the majority of which were cross-sectional, indicated that poor sleep quality or disturbance is positively associated with SI. Undisputedly, perinatal sleep, fatigue, and depressive symptoms have reciprocal influences (78). On the one hand, sleep disturbances are an immediate sequela of depression and anxiety (79), and on the other hand, poor sleep is a risk factor for mood disorders (80) and SIB (81). Thus, exploring these relationships over time is crucial. Interventions targeting insomnia symptoms, for example, were found to contribute to reducing symptoms of depression among women in the third trimester of pregnancy (82). The high incidence of sleep disturbances among pregnant and postpartum women highlight the critical importance of further research in this field and the need to make medical, treatment, and research professionals.

As for variables that may moderate or mediate the relationship between sleep disorders and suicidal ideation the findings indicate the possible influence of various psychosocial factors on the relationship between sleep problems and SIB. In a systematic review it was found, for example, that thwarted belongingness or social isolation may explain the relationship between sleep and SIB (83). It can be assumed that similar relationships may also be found among perinatal women, given the importance of social support during the perinatal period combined with the lack of quality sleep among most women during this period. Therefore, research on those associations in perinatal period is imperative.

Medical condition of pregnant and postpartum women or those around them was found to be another possible risk factor for perinatal SIB, which, to our knowledge, was not considered in previous reviews. In this review only seven studies focused on this risk factor and it seems that research in this area is still lacking. In general, it was found that a history of chronic illness is a risk factor for SIB during pregnancy. However, almost all of the studies reviewed here investigated this aspect in reference only to HIV-related outcomes. It was found that high stigma or shame resulting from these women’s condition was a risk factor for SI during the perinatal period (38, 39). In addition, among pregnant women, an association between migraines and SI was found (42). In the present review only one study addressed the impact of the newborn’s medical condition on the mother’s mental state. The findings suggested a link between the newborn’s illness and the mother’s SB (43). These findings further reinforce the vigilance and attention, in terms of research and clinical practice, which should be given to perinatal woman’s various medical conditions and her environment and to their potential for a negative emotional impact to the point of suicidal distress. It is also important to keep in mind that each medical condition or disease has unique characteristics and therefore different effects on the person dealing with them. In light of this, specific studies that pertain to the unique characteristics of each medical condition and their influence on perinatal women in general and suicide ideations and behavior in particular, are necessary.

Some studies investigated interpersonal factors, mainly social support. It was found in most reviewed studies that high social support is a resilience factor for suicidal risk among perinatal women. Interestingly, in one longitudinal study, perceived family support was only related to SI severity at 4-month postpartum (52). This finding reinforces the importance of further longitudinal studies in the field, mainly to deepen the understanding of what is the most critical time to support a woman during this period. From a theoretical point of view, Joiner’s Interpersonal Theory of Suicide emphasizes the strong link between interpersonal relationships and suicidality. According to this theory, suicidal desire results from the convergence of two interpersonal states: perceived burdensomeness and thwarted belongingness, and that SB occur in the presence of suicidal desire and capability for suicide (84–86).

For example, a woman who suffers from PPD and has difficulty caring for her baby on her own may feel a burden on her environment and a lack of belonging on several levels and be at increased risk of suicide.

In this review, it was also found that an unsafe attachment style among pregnant women or mother-child bonding impairment among postpartum women may be potential risk factors as well. This aspect has not been extensively researched and may be critical in identifying peripartum women who are in distress but do not report it directly.

As noted in almost all studies, psychopathology was a significant risk factor for SIB during the perinatal period. Depression, anxiety, and history of SIB mainly identified as associated with current suicidal risk. Other psychiatric disorders, such as bipolar disorder, borderline personality, PTSD and nocturnal rumination, were also related in some studies. In addition, some studies found an association between smoking (direct or passive) and alcohol use and suicidal risk among pregnant and postpartum women (33, 51).

Furthermore, although many of the studies naturally emphasized depression as a major risk factor for suicidality, some of perinatal women with SI did not necessarily suffer from depression (45, 70). These results highlight the importance of assessing symptoms of depression and suicide separately based on the assumption that besides depression, there are other clinical phenomena independently associated with SIB.

One variable which was recently studied in the context of SIB (87, 88) and that may be relevant to women in the perinatal period is the concept of demoralization, defined as “a state of self-perceived incapacity to act at some minimal level according to some internalized standard in a specific stressful situation” (89). There is an overlap between depression and demoralization, but nevertheless, there are fundamental differences. For example, a central component of depression is anhedonia, while in demoralization, it is losing one’s sense of effectiveness (87). Further investigation of this concept may shed light on the plight of suicidal but not depressed perinatal women.

The last significant risk factor in the current literature review was aversive life events. All reviewed studies reported a positive association between such experiences and suicidal risk among pregnant or postpartum women. Some addressed aversive childhood events (e.g., childhood abuse, CM), while others focused on aversive events in adulthood, the majority of which investigated IPV. Most forms of domestic violence were significantly associated with SI in pregnancy and postpartum. Life events in themselves can have an immediate and long-term effect in stressful situations (90). Since childbirth is a stressful event in itself, it is imperative to identify those women at risk, especially women with serval stressful life events combined with existing psychopathology, interpersonal difficulties, and sleep disturbance.

### Methodological limitations of the study of suicidality among pregnant and postpartum women

One of the most significant limitations of research in this field is the use of tools designed to assess depression to identify suicidal risk. Mainly used is the Edinburgh Postnatal Depression Scale (EPDS) (27) and the Patient Health Questionnaire (PHQ-9) (91) as shown in Table 1. In both, the last question (tenth in EPDS and ninth in PHQ-9) refers to SI. Although this limitation is known and mentioned in some of the studies presented in this review and in some previous reviews, these tools are still used extensively to assess suicidal risk in research and the clinical field. Beyond the problematic use of only one question to assess suicidal risk, additional problems arise: along with the strong relationship between depression and suicide in general, and specifically, during the perinatal period, there are women who, despite being at risk for suicide, are not depressed and therefore may be overlooked when using these tools.

Another limitation is the lack of longitudinal research in the field. As mentioned above, the professional terminology was impacted over a decade ago by the growing knowledge of the strong connection between the woman’s mental state during pregnancy and postpartum. Indeed, in 2013, the American Psychiatric Association (APA) modified the terminology from postpartum depression to peripartum depression, based on the recognition that a woman’s mental distress may begin as early as pregnancy and not only after childbirth (8). Nevertheless, and probably due to the resources required for longitudinal research, most studies are cross-sectional and thus artificially separate the periods, thereby losing much information about the variability of distress over time.

A further limitation is related to the types of SIB included in this review. To investigate various risk factors in-depth, the present review included sample studies only and did not include epidemiological studies or studies based on institutional databases in which data and frequencies on active SA often appear. As a result, most of the studies included in the current review examined SI and scarcely addressed SB.

It is also worth noting that about 50% of the studies in this review were conducted in low- and mid-income countries or among women of weakened socioeconomic status. This percentage indicates an increase in research attention in these countries to suicide among perinatal women and perhaps also points to a decrease in research in high-income countries or among women of a high socioeconomic status. There is a risk of suicide among pregnant and postpartum women worldwide, and cultural and social diversity requires a culturally sensitive research approach. To the best of our knowledge, cultural and social aspects have hardly been explored in the literature, and therefore it is vital to deepen future research related to these issues.

Finally, due to the significant differences between studies in terms of study design, research tools, and sample types, it was impossible to merge the data and perform a meta-analysis or quality assessment.

## Conclusion and clinical implications

As accumulated research and clinical knowledge suggest, disturbed sleep plays a dominant risk factor for SIB among women during the perinatal period. However, so far sleep disturbance has not been studied in depth as a significant risk factor during this period. One of the possible reasons lies in the fact that almost all pregnant or postpartum women will experience varying degrees of disturbed sleep. This may prevent family members, medical professionals as well as the woman herself from recognizing the effect sleep difficulties have on her mental health in general and her risk for suicidal behavior in particular. The results emphasize the importance of regular screening for sleep disturbance, during pregnancy and throughout the first year after birth. Screening can be carried out by nurses in prenatal and postnatal clinics, or by other support factors who are in constant contact with women at this stage.

The findings also highlight the importance of a support network for pregnant and postpartum women. It is important to carry out comprehensive screenings in order to understand the women’s objective and subjective support systems, since the research literature indicates that sometimes perceived social support is even more important than objective support.

Initiating treatment or prevention programs during the prenatal period among women at risk for suicide, may play a vital role in preventing depression and risk for suicide during the postpartum period. It is of critical importance to provide psychoeducation to those particular women so it can help them and their immediate environment identify various causes of distress, as well as provide them information regarding appropriate support systems in the community.

In addition, the need to detect SIB separately from depressive symptoms requires implementing tools specifically designed to assess SIB in pregnancy and childbirth follow-up clinics.

Last but not least, medical and psychological staff need to undergo periodic training on the variety of risk factors for depression and suicide among perinatal women. In addition, acquaintance with the diversity and evidence based therapeutic interventions may help professionals identify and prevent aggravation of suicidal behavior among perinatal women.

## Data availability statement

The original contributions presented in this study are included in this article/supplementary material, further inquiries can be directed to the corresponding author.

## Author contributions

BA-A: data collection and analysis and writing—original draft preparation. YG and SH: conceptualization, supervision, and editing. MW: data collection. All authors contributed to the article and approved the submitted version.
